# Recent Toxoplasmosis Infection With Acute Myopericarditis and Persistent Troponin Elevation in an Immunocompetent Patient

**DOI:** 10.4021/cr200w

**Published:** 2012-07-20

**Authors:** François Roubille, Camille Roubille, Benoît Lattuca, Richard Gervasoni, Hélène Vernhet-Kovacsik, Florence Leclercq

**Affiliations:** a CHU Arnaud de Villeneuve, Cardiology Department, Montpellier, France; b CHU Saint Eloi, Internal Medicine Department, Montpellier, France; c CHU Arnaud de Villeneuve, Vascular Radiology Department, Montpellier, France

**Keywords:** Toxoplasmosis, Acute myocarditis, Myopericarditis, MR-scan

## Abstract

Although often considered as "begnin", acute infections in young healthy adults can lead to heart inflammation, including acute myocarditis. We report a rare case of myopericarditis in a young immunocompetent adult, in the context of recent toxoplasmosis infection. Clinical presentation was common acute pericarditis, but with risk biomarkers: high troponin I levels and multiple inflammation-compatible images on MR-scan. Diagnosis of myopericarditis was established. In spite of spontaneous favourable clinical evolution, troponin remained elevated. MR-scan is shown; acute myocarditis in the context of an acute toxoplasmosis infection is discussed.

## Introduction

In prospective studies among pericarditis, toxoplasmosis is rarely described [1]. Besides, acute myopericarditis by toxoplasma in immunocompetent patients has been rarely reported. Here, we describe for the first time acute myopericarditis in a young immunocompetent patient and present the MR findings.

## Case Report

A 24-year-old man was admitted for acute chest pain which prevented him from breathing normally. He had no previous medical history, no cardiovascular risk factors and was not on any medications. He had never travelled, had no recent infectious context or viral syndrome. On admission the patient was hemodynamically stable, no febrile. Physical examination didn’t reveal pericardial rub, heart failure. Neither hepatomegaly nor splenomegaly nor lymph nodes were palpable. A chest X-ray was normal. ECG showed a normal sinus rhythm,and a mild ST-segment elevation, especially in V2-V3, without AV-block. Echocardiography was strictly normal, without pericardial effusion, nor any other abnormalities. The day after, fever appeared (38 °C), new echocardiography revealed a mild pericardial effusion (3 mm).

Red cell count and hemoglobin level were normal, white cell count showed polynucleosis (11,000 cells/mm^3^, N < 10,000) and monocytosis (1,476, N < 1,000). Inflammation biomarkers were increased (initial CRP = 18 mg/L, the day after 24, N < 5 mg/L; procalcitonin remained normal under 0.5 ng/mL). Liver enzymes were not elevated. In addition, troponin I increased and peaked at 17.4 µg/L on the second day after admission (N < 0.05). Several months later, troponin remained elevated, among 0.5.

### Etiologic examination

All of the initial etiologic studies remained negative or non-specific: CMV, EBV, parvovirus B19, HBV, HCV, HIV1, 2 *bartonella, borrelia, chlamydiae, coxiella, mycoplasma, rickettsia*. TSH was normal. Plasmatic immuno-electrophoresis was normal. Anti-DNA antibodies were absent, hemostasis was normal.

### MR-scan

**Figure 1:**
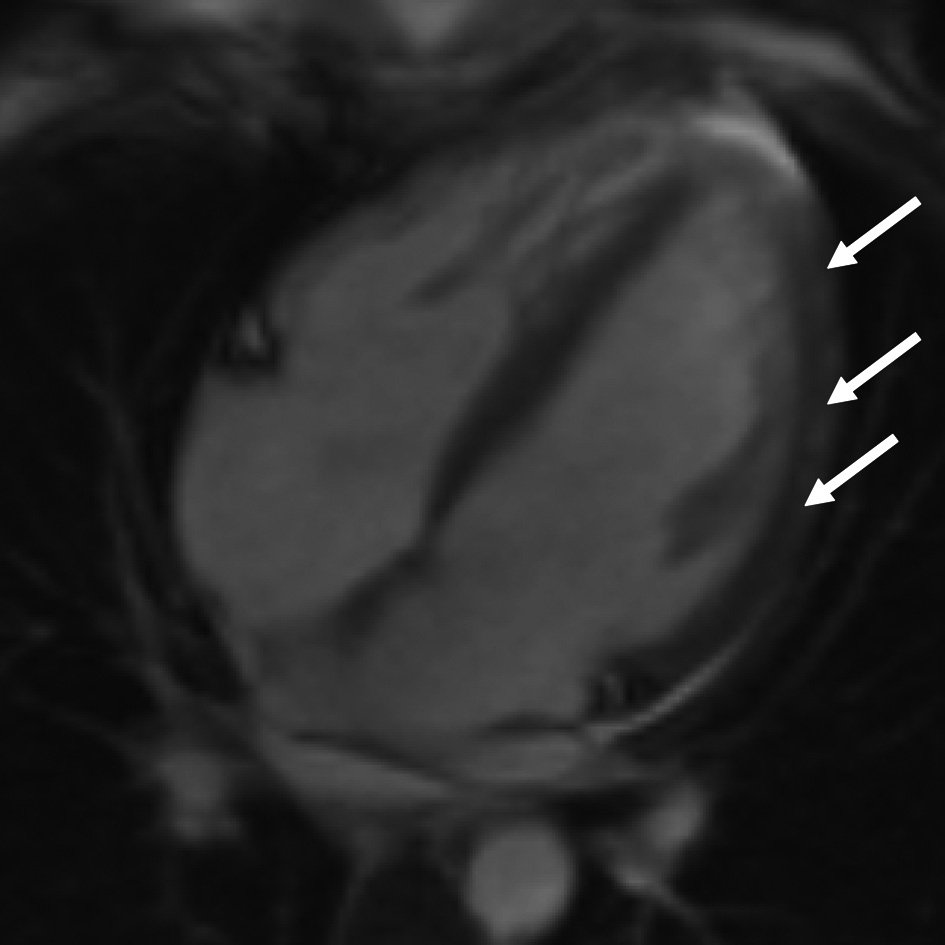
Cardiac MRI. Delayed-enhancement sequences performed after perfusion of gadolinium demonstrated a diffuse lumpy subepicardial enhancement (white arrows).

The scanner (1.5 T Avento MR scanner, Siemens Medical Systems, Erlangen, Germany) was performed three days later, and is presented in Figure 1. Cardiac function was normal, with no focal alteration of the contractility, and a left ventricular ejection fraction was estimated at 52%. Delayed enhancement sequences, performed after perfusion of gadolinium demonstrated a lumpy subepicardial diffuse enhancement. Mild pericardial effusion was confirmed. These findings were concordant with the diagnosis of myocarditis (Fig. 1).

### Serial etiologic study

Serological evaluation for toxoplasmosis showed recent acute infection: IgM titres to *Toxoplasma gondii*, were 0.848 IU/mL (International Unit) (positive ≥ 0.5); and IgG titres were 10 IU/mL, and one month later 340 IU/mL (positive ≥ 5).

No myocardial biopsy was performed.

The patient was discharged a few days later, under the following DAILY treatment: aspirin 3 g, colchicin 1 mg, bisoprolol 2.5 mg, esomeprazole 20 mg, per day.

One month later, he felt very well, and presented none of the previous symptoms. Nevertheless, troponin remained elevated (0.4 µg), and serological examinations claimed for toxoplasmosis, so that we wanted to discuss RMI revaluation, cerebral scan, and specific treatment.

Unfortunately, the patient refused, and no specific treatment was given.

## Discussion

In immunocompromised patients, especially in HIV patients, toxoplasmosis can lead to severe cardiac disorders [2, 3]. In immunocompetent patients, toxoplasmosis infections induce only exceptional cardiac symptoms, but could be underdiagnosed or only at dilated state [4] and could even lead to sudden death [5]. Clinical cases have been scarcely reported. Most often, myocarditis is described without MR-scan images nor histology [6], or numerous cardiovascular risk factors raise doubt about coronary disease. Clinical case in a young healthy adult is reported and heart involvement is supposed on account of atrioventricular block [7].

Although diagnosis cannot but be supposed, myocardial biopsy is not usually performed, on account of lethal risk. To our knowledge, it is the first time serological conversion for toxoplasmosis, with cardiac involvement evidenced on MR-scan, and biological myocardial enzymes elevation, is reported. This is of great importance as enzymes elevation could be linked with poor outcomes, urging to a stricter follow-up of these patients [8].

Antibiotics are to be discussed. Unfortunately, this healthy patient refused every treatment. Cardiac involvement has been scarcely reported, so that natural evolution is not known. In our case, clinical outcome was quickly good during hospitalization, and follow-up confirmed total clinical recovery, although active myocardial involvement could be evoked. Interestingly, interest of rest had to be extensively discussed with the patient, as this raised recently as a pivotal tool to obtain heart rate lowering [9].

### Conclusion

As far as we know it is the first time acute myocarditis linked to a recent toxoplasmosis infection is reported, with consistent MR-scan images. Clinically significant heart injury may be a rare, but life-threatening, manifestation of toxoplasmosis.

## References

[1] Roubille F, Roubille C, Rullier P, Saada M, Cayla G, Macia JC, Piot C (2008). [Daily management of acute pericarditis: clinical and paraclinical outcomes, etiological diagnosis]. Ann Cardiol Angeiol (Paris).

[2] Eza DE, Lucas SB (2006). Fulminant toxoplasmosis causing fatal pneumonitis and myocarditis. HIV Med.

[3] Chimenti C, Del Nonno F, Topino S, Abbate I, Licci S, Paglia MG, Capobianchi MR (2007). Fatal myocardial co-infection by Toxoplasma gondii and Parvovirus B19 in an HIV patient. AIDS.

[4] Dobranici M, Buzea A, Popescu R, Chirila L (2012). Genetic disorder or toxoplasma myocarditis: a case report of dilated cardiomyopathy with hypertrabeculation in a young asymptomatic woman. J Med Life.

[5] Lanjewar DN, Agale SV, Chitale AR, Joshi SR (2006). Sudden death due to cardiac toxoplasmosis. J Assoc Physicians India.

[6] Chandenier J, Jarry G, Nassif D, Douadi Y, Paris L, Thulliez P, Bourges-Petit E (2000). Congestive heart failure and myocarditis after seroconversion for toxoplasmosis in two immunocompetent patients. Eur J Clin Microbiol Infect Dis.

[7] Mroczek-Czernecka D, Rostoff P, Piwowarska W (2006). [Acute toxoplasmic perimyocarditis in a 67-year-old HIV-negative woman—a case report]. Przegl Lek.

[8] Machado S, Roubille F, Gahide G, Vernhet-Kovacsik H, Cornillet L, Cung TT, Sportouch-Dukhan C (2010). Can troponin elevation predict worse prognosis in patients with acute pericarditis?. Ann Cardiol Angeiol (Paris).

[9] Ziad Khoueiry CR, Nicolas Nagot, Benoît Lattuca, Florence Leclercq, Delphine Delseny, David Busseuil, Richard Gervasoni, Christophe Piot, Jean-Marc Davy, Jean-Luc Pasquié, Frédéric Cransac, Catherine Sportouch-Dukhan, Jean-Christophe Macia, Thien-Tri Cung, François Massin, Stéphane Cade, Jean-Paul Cristol, François Roubille (2012). Could heart rate play a role in pericardial inflammation?. Medical Hypothesis.

